# Insights into the Evolution of Aphid Mitogenome Features from New Data and Comparative Analysis

**DOI:** 10.3390/ani12151970

**Published:** 2022-08-03

**Authors:** Hui Zhang, Congcong Lu, Qian Liu, Tianmin Zou, Gexia Qiao, Xiaolei Huang

**Affiliations:** 1 Key Laboratory of Zoological Systematics and Evolution, Institute of Zoology, Chinese Academy of Sciences, Beijing 100101, China; zhanghui1903@163.com; 2 State Key Laboratory of Ecological Pest Control for Fujian and Taiwan Crops, College of Plant Protection, Fujian Agriculture and Forestry University, Fuzhou 350002, China; lcchuaer@163.com (C.L.); liuqian9502@163.com (Q.L.); zou1208986257@163.com (T.Z.)

**Keywords:** control region, gene rearrangement, insect, mitochondrial genome, phylogeny, repeat region

## Abstract

**Simple Summary:**

Complete mitogenomes provide useful information for investigating the molecular evolution and phylogenetic relationships of insects. This paper reports three new aphid mitogenomes, and provides comparative genomic analyses to investigate the evolution of some unique features of aphid mitogenomes. All three mitogenomes contain the unique tandem repeat region and exhibit notably rearranged gene orders. We provide new support for the idea that tandem repeat region is an ancient feature of aphid mitogenomes, and also demonstrate that the discovered striking gene rearrangements in some aphid mitogenomes are independent clade-specific evolutionary events at certain taxonomic levels. This study improves our understanding of aphid mitogenome evolution.

**Abstract:**

The complete mitochondrial genomes and their rearrangement patterns can provide useful information for inferring evolutionary history of organisms. Aphids are one of the insect groups with some unique mitogenome features. In this study, to examine whether some features in aphid mitogenomes are independent species-specific evolutionary events or clade-specific events at certain taxonomic levels, we sequenced three new aphid mitogenomes (Hormaphidinae: *Ceratovacuna keduensis*, *Pseudoregma panicola*; Lachninae: *Nippolachnus piri*) and compared them with all known aphid mitogenomes. The three mitogenomes are 16,059–17,033 bp in length, with a set of 37 typical mitochondrial genes, a non-coding control region and a tandem repeat region. The gene orders of them are all highly rearranged. Within the subfamily Hormaphidinae, the presence of repeat region and mitogenome rearrangement in Cerataphidini species but not in the other two tribes indicate that these may be Cerataphidini-specific features. The same gene rearrangement pattern in the two Lachninae species, *N. piri* (Tuberolachnini) and *Stomaphis sinisalicis* (Stomaphidini), supports that this feature should be at least derived from the common ancestor of two tribes. Overall, our data and analyses provide new insights into the evolutionary patterns of gene rearrangement and repeat region in aphid mitogenomes, and further corroborate the potential role of gene rearrangement in elucidating the evolutionary history of different insect lineages.

## 1. Introduction

The genomes of mitochondria are double-strand and highly conserved circular molecules with genes required for many biological processes such as energy transduction, metabolism, and apoptosis [1,2]. Insect mitochondrial genomes (mitogenomes) are typically composed of 37 genes, including 13 protein-coding genes (PCGs), two ribosomal RNAs (rRNAs), and 22 transfer RNAs (tRNAs). In addition, there are also some non-coding regions in insect mitogenomes, such as the A + T-rich control region (CR), short intergenic spacers, and repeat region existing in some particular insect taxa, such as the tandem repeat region locating between *trnE* and *trnF* in some aphid mitogenomes. The gene arrangement within insect mitogenomes is generally conserved across different taxonomic groups and identical to that of inferred ancestral insect gene order [3,4]. However, with the increasing availability of complete mitogenomes, different types of gene rearrangements including gene transposition, gene inversion, gene duplication, and gene loss, etc., are discovered in many insect taxonomic groups covering species from different orders such as Hymenoptera [5,6], Thysanoptera [7], Psocoptera [8], Phthiraptera [9,10], and Hemiptera [11]. Although the underlying mechanisms for these mitogenome rearrangements are currently unclear, several possible models have been proposed to account for these different types of rearrangement events, including the tandem duplication random loss (TDRL) model [12,13], tandem duplication and non-random loss (TDNL) model [13], and recombination model [14]. Gene rearrangements in the insect mitogenomes can offer great potential for phylogenetic studies [15,16], and the comparison of rearrangement patterns across different lineages can also promote better understanding of mitogenome evolution and evolutionary history of insects.

Among species-rich hemipteran insects, mitochondrial genome rearrangements mostly occur in planthoppers [17], true bugs [18,19], whiteflies [11], and scale insects [20,21]. Nevertheless, aphids represent a diverse group of hemipterans comprising many economically important agricultural pests, exhibiting a relatively conserved ancestral mitogenome arrangement in most species. To date, more than fifty complete mitogenomes covering eight subfamilies have been determined and annotated in aphids (Table S1), representing a very limited resource relative to the total aphid species richness. A paucity of mitogenome data from aphids has precluded investigations on mitogenome evolution among lineages representing different taxonomic levels. Based on the currently available aphid mitogenomes, gene rearrangement has only been discovered in species from several different clades, including *Pseudoregma bambucicola* (Hormaphidinae: Cerataphidini), *Stomaphis sinisalicis* (Lachninae: Stomaphidini) and some species from Fordini (Eriosomatinae) and Aphidinae [22]. In Fordini, mitogenome rearrangement involving transposition of *trnQ* and *trnM* has been found in all species with sequenced mitogenomes, with the exception of the basal species *Baizongia pistaciae* [22,23,24]. Information about mitogenome annotations available via the GenBank database indicates that gene rearrangements have occurred in some Aphidinae species [22]; however, this may be incorrect due to unreliable annotations by original data submitters (see our new analysis below). Furthermore, gene rearrangements involving five tRNAs and one PCG are also found in *P. bambucicola* from Cerataphidini, while two Hormaphidini species from the same subfamily show a putative ancestral insect gene order [22,25]. This pattern has raised the question as to whether this gene rearrangement is an independent evolutionary event of *P. bambucicola* or a common evolutionary feature for all Cerataphidini species. The lack of complete mitogenomes of other Cerataphidini species has limited our understanding of the real evolutionary history of mitogenome rearrangement within this clade. Moreover, the mitochondrial gene order of a Lachninae species *S. sinisalicis* is also highly rearranged in comparison with that of other aphids, and exhibits a unique gene arrangement pattern [22]. However, based on a single species, it is also difficult to draw a definite conclusion regarding whether this distinct arrangement pattern is a species-specific evolutionary event for *S. sinisalicis* or a shared evolutionary feature for Lachninae species. Therefore, the accumulation of more evidence from broader sampling or a wider spectrum of taxonomic groups is necessary to verify these assumptions. 

Here, we have newly sequenced and determined mitogenomes of three aphid species, including *Ceratovacuna keduensis* and *Pseudoregma panicola* from Cerataphidini (Hormaphidinae) and *Nippolachnus piri* from Tuberolachnini (Lachninae), to further explore the evolutionary context of previously revealed striking rearrangement patterns in *P. bambucicola* and *S. sinisalicis*. The general organization and the gene order of the three mitogenomes were analyzed and compared with that of other available aphid mitogenomes to investigate the evolution of tandem repeats and gene arrangement patterns across different aphid lineages. These mitogenomes will provide valuable data resources for studying the evolution of mitogenomes. Moreover, a comprehensive investigation of gene arrangement patterns in these aphid mitogenomes will facilitate better understanding of phylogenetic relationships and evolutionary history of different aphid lineages.

## 2. Materials and Methods

### 2.1. Sample Collection and DNA Isolation

Samples of *C. keduensis*, *P. panicola*, and *N. piri* were collected on bamboo, *Cyrtococcum patens* and *Pyrus*, from Fujian Province, China, in September 2017, December 2016 and May 2016, respectively. All specimens and vouchers were preserved in 95% alcohol at −80 °C at the Insect Systematic and Diversity Lab at Fujian Agricultural and Forestry University, Fuzhou, China. Genomic DNA was extracted from female adults of each species using DNeasy Blood &Tissue Kit (QIAGEN, Hilden, Germany) according to the manufacturer’s instructions, and the DNA concentration was measured using a Qubit fluorimeter (Invitrogen, Carlsbad, CA, USA).

### 2.2. Mitogenome Sequencing, Assembly and Annotation

Quantified genomic DNA was used for library construction using TrueLib DNA Library Rapid Prep Kit (ExCell Bio, Shanghai, China), and then paired-end sequencing (2 × 150 bp reads) was performed on an Illumina NovaSeq platform with an average insert size of 350 bp. A total of 10.3 Gb, 10.2 Gb and 10.4 Gb of raw data were generated for *C. keduensis*, *P. panicola*, and *N. piri*, respectively. The raw sequencing data were pre-processed using Trimmomatic v0.35 [26] to remove adapters and low-quality reads to obtain a high-quality clean data. Finally, 8.8 Gb, 8.8 Gb, and 8.9 Gb clean data were used for mitogenome assembly for *C. keduensis*, *P. panicola*, and *N. piri*, respectively. Mitogenomes for three aphid species were assembled with NovoPlasty v2.7.1 [27] with default parameters, using the *cox1* sequence of each species as the seed sequence. 

The MITOS web server [28] was used for annotation of three newly sequenced mitogenomes. PCGs were further determined by their open reading frames based on the invertebrate mitochondrial genetic code and alignment with homologous genes of other aphids (Table S1). The tRNAs were annotated using the MITOS web server and the program ARWEN v1.2 [29], and their secondary structures were further predicted by RNAplot using the ViennaRNA Package [30]. Two rRNAs were identified by alignment with other aphid rRNA sequences. The control regions were determined through the boundaries of adjacent genes. Tandem repeat regions in each aphid mitogenome were detected using the Tandem Repeats Finder Web server (http://tandem.bu.edu/trf/trf.html (accessed on 3 November 2021)) [31]. Nucleotide skew values were calculated using the formulae AT-skew = (A − T)/(A + T) and GC-skew = (G − C)/(G + C) [32]. CGView Server [33] was used for generating the circular maps of three aphid mitogenomes. The non-synonymous substitutions (Ka) and synonymous substitutions (Ks) for 13 PCGs were calculated in DnaSP v6.12.03 [34]. The evolutionary rates for PCGs were evaluated using Ka/Ks ratios, which are used for inferring different types of selective pressures acting on genes, with the Ka/Ks < 1 indicating purifying selection, Ka/Ks = 1 indicating neutral selection and Ka/Ks > 1 indicating positive selection [35].

### 2.3. Phylogenetic Analysis and Evolutionary Pattern of Tandem Repeats

Phylogenetic analysis was conducted using PCGs from three species in this study and other aphid species with available complete mitogenomes, using *Adelges tsugae* as the outgroup. Nucleotide sequences of 13 PCGs were individually aligned under codon-based alignment mode using MAFFT v7.149 [36] with default parameters. All the aligned sequences were translated into amino acids and sequence regions that were not aligned properly from both ends were trimmed. PhyloSuite v1.2.1 [37] was then used to generate a concatenated sequence of 13 alignment datasets. The concatenated sequence was then used for maximum-likelihood (ML) tree estimation with IQ-TREE [38] integrated in PhyloSuite v1.2.1 under the automatic predicted GTR + F + I + G4 model using SH-aLRT test with 5000 ultrafast bootstraps.

To further explore the distribution and diversity of tandem repeats between *trnE* and *trnF* and that inserted into the control regions across different aphid lineages, the mitogenomes of *C. keduensis*, *P. panicola* and *N. piri*, and known complete mitogenomes of other aphids were further detected for tandem repeat sequences with the Tandem Repeat Finder. The tandem repeats between *trnE* and *trnF* or within the control region were then mapped onto the phylogeny of aphids to examine their evolutionary pattern across different lineages.

### 2.4. Gene Arrangement Analysis

The gene orders of all known aphid mitogenomes were characterized and compared with ancestral insect gene order to investigate mitogenome rearrangement patterns in aphids under a phylogenetic framework. Specifically, PhyloSuite v1.2.1 [37] was used for extracting gene order files of aphid mitogenomes based on their GenBank accession numbers (Table 1). The online tool Interactive Tree of Life (iTOL) [39] was then used for the visualization of gene arrangement patterns of aphid mitogenomes, with the gene order files mapping onto the aphid phylogeny. The CREx tool [40] was used for comparisons of pairwise gene orders and inferring rearrangement events that have occurred in mitogenomes of each aphid species using the arrangement pattern of *Drosophila yakuba* mitogenome as the referential ancestral insect gene order [41]. Notably, in order to ensure the accuracy and reliability of gene rearrangement analysis, known aphid mitogenomes were reannotated and the rearranged genes were further verified by comparing with homologous genes of related species. Calibrated gene order for each aphid species was used for the final comparative analysis of mitogenome arrangement patterns. Members of the same tribe with identical gene order randomly retained only one representative species to display on the final figure.

## 3. Results

### 3.1. General Organization of Three Aphid Mitogenomes

**Figure 1:**
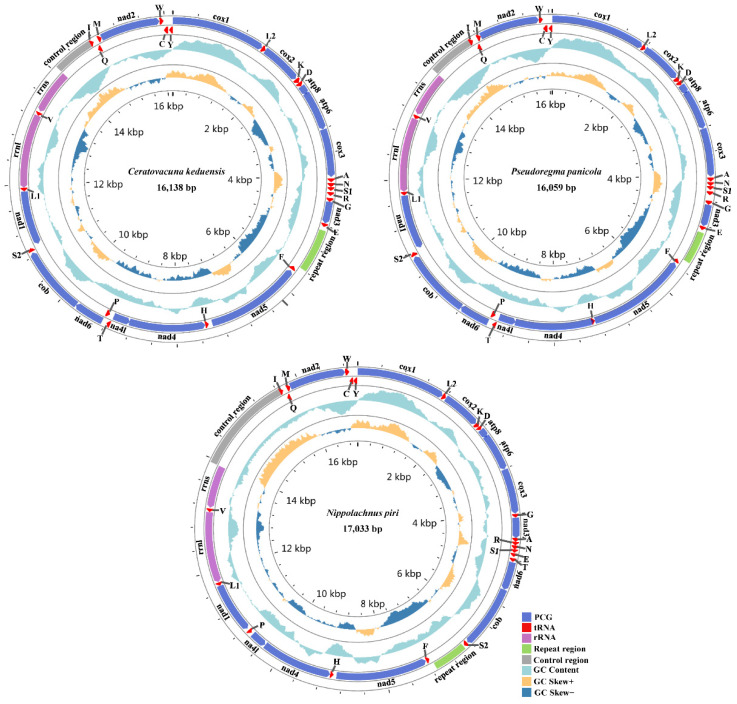
Circular maps of mitogenomes for *Ceratovacuna keduensis*, *Pseudoregma panicola*, and *Nippolachnus piri*. Genes or gene regions are highlighted by different colors. The tRNAs are indicated by single-letter amino acid codes. The outermost circle shows the gene arrangement with arrows indicating the direction of transcription. The second circle indicates the GC content with the celeste shading above and below denotes GC content values greater than and less than the genome average, respectively. The third circle indicates the GC skew with the light orange shading above, and indigo blue shading below denotes GC skew values greater or less than the genome average, respectively. The innermost circle with scale shows nucleotide position on the genome.

The complete mitogenomes of *C. keduensis* and *P. panicola* have a size of 16,138 bp and 16,059 bp, respectively, which are comparable to that of another Cerataphidini species *P. bambucicola* (16,632 bp). In contrast, *N. piri* has a larger mitogenome than *C. keduensis*, *P. panicola* and most other aphids, which is 17,033 bp in length and similar to that of *S. sinisalicis* (17,109 bp) from the same subfamily Lachninae (Table 1 and Table S1). The circular maps for three mitogenomes are shown in Figure 1. All three aphid mitogenomes have a typical set of 37 insect mitochondrial genes, including 13 PCGs, 2 rRNAs, and 22 tRNAs. Of these genes, 14 tRNAs and 9 PCGs are encoded on the major strand (J-strand), and the other 14 genes, including 8 tRNAs, 4 PCGs, and 2 rRNAs are encoded on the minor strand (N-strand) (Tables S2–S4). There is also a non-coding control region and a tandem repeat region in all three mitogenomes. As with all other aphid species, the three newly sequenced mitogenomes also exhibit strong AT bias, with A + T contents of 84.9%, 85.0%, and 83.9% for *C. keduensis*, *P. panicola*, and *N. piri*, respectively. All three mitogenomes present a positive AT skew and a negative GC skew (Table 2), which is congruent with other known aphid mitogenomes [22,42].

**Table 1:** Information of complete aphid mitogenomes used in this study.

Subfamily	Tribe	Species	Length (bp)	Accession Number	Reference
Outgroup		*Adelges tsugae*	16,056	MT263947	[43]
Lachninae	Stomaphidini	*Stomaphis sinisalicis*	17,109	NC_053790	[22]
	Tuberolachnini	*Nippolachnus piri*	17,033	OL069343	This study
Hormaphidinae	Cerataphidini	*Ceratovacuna keduensis*	16,138	OL069341	This study
	Cerataphidini	*Pseudoregma bambucicola*	16,632	NC_044640	[25]
	Cerataphidini	*Pseudoregma panicola*	16,059	OL069342	This study
	Hormaphidini	*Hamamelistes spinosus*	15,089	MT010853	[44]
	Hormaphidini	*Hormaphis betulae*	15,088	NC_029495	[45]
	Nipponaphidini	*Schizoneuraphis gallarum*	14,990	NC_053624	[46]
Eriosomatinae	Eriosomatini	*Eriosoma lanigerum*	15,640	NC_033352	[47]
	Eriosomatini	*Paracolopha morrisoni*	16,330	NC_045103	[48]
	Fordini	*Baizongia pistaciae*	15,602	NC_035314	[23]
	Fordini	*Floraphis choui*	15,308	NC_035310	[49]
	Fordini	*Floraphis meitanensis*	15,301	NC_035316	[49]
	Fordini	*Kaburagia rhusicola ensigallis*	16,164	MF043984	[23]
	Fordini	*Kaburagia rhusicola ovatirhusicola*	16,184	MF043985	[23]
	Fordini	*Kaburagia rhusicola ovogallis*	16,164	MF043986	[23]
	Fordini	*Kaburagia rhusicola rhusicola*	16,159	MF043987	[23]
	Fordini	*Meitanaphis elongallis*	16,191	NC_035315	[49]
	Fordini	*Meitanaphis flavogallis*	16,150	NC_035312	[49]
	Fordini	*Meitanaphis microgallis*	16,191	NC_047419	[50]
	Fordini	*Melaphis rhois*	15,436	NC_036065	[51]
	Fordini	*Nurudea ibofushi*	16,054	NC_035311	[23]
	Fordini	*Nurudea shiraii*	15,389	NC_035301	[23]
	Fordini	*Nurudea yanoniella*	15,858	NC_035313	[49]
	Fordini	*Schlechtendalia chinensis*	16,047	NC_032386	[52]
	Fordini	*Schlechtendalia peitan*	15,609	NC_035302	[23]
Greenideinae	Cervaphidini	*Cervaphis quercus*	15,272	NC_024926	[53]
	Greenideini	*Eutrichosiphum pasaniae*	16,500	NC_054157	[54]
	Greenideini	*Greenidea ficicola*	17,361	NC_048525	[55]
	Greenideini	*Greenidea psidii*	16,202	NC_041198	[56]
	Greenideini	*Mollitrichosiphum tenuicorpus*	15,727	NC_054348	[57]
	Schoutedeniini	*Schoutedenia ralumensis*	16,051	MT381994	[58]
Chaitophorinae	Chaitophorini	*Periphyllus diacerivorus*	16,418	MZ665537	Direct submission
Calaphidinae	Panaphidini	*Appendiseta robiniae*	15,049	NC_042165	[59]
	Panaphidini	*Therioaphis trifolii*	16,068	MK766411	[60]
Aphidinae	Aphidini	*Aphis aurantii*	15,469	MN397939	[61]
	Aphidini	*Aphis citricidus*	16,763	NC_043903	[24]
	Aphidini	*Aphis craccivora*	15,308	NC_031387	[62]
	Aphidini	*Aphis fabae mordvilkoi*	15,346	NC_039988	[59]
	Aphidini	*Aphis glycines*	17,954	NC_045236	[63]
	Aphidini	*Aphis gossypii*	15,869	NC_024581	[64]
	Aphidini	*Aphis spiraecola*	16,500	NC_053819	[65]
	Aphidini	*Hyalopterus pruni*	15,410	NC_050904	[66]
	Aphidini	*Melanaphis sacchari*	15,111	MW811104	Direct submission
	Aphidini	*Rhopalosiphum nymphaeae*	15,594	NC_046740	[67]
	Aphidini	*Schizaphis graminum*	15,721	NC_006158	[11]
	Macrosiphini	*Acyrthosiphon pisum*	16,971	NC_011594	Direct submission
	Macrosiphini	*Brevicoryne brassicae*	15,927	NC_056270	[68]
	Macrosiphini	*Cavariella salicicola*	16,317	NC_022682	[42]
	Macrosiphini	*Chaetosiphon fragaefolii*	16,108	LC590896	[69]
	Macrosiphini	*Diuraphis noxia*	15,784	NC_022727	[70]
	Macrosiphini	*Indomegoura indica*	15,220	NC_045897	[71]
	Macrosiphini	*Myzus persicae*	17,382	NC_029727	[59]
	Macrosiphini	*Neotoxoptera formosana*	15,642	MW534268	[72]
	Macrosiphini	*Sitobion avenae*	15,180	NC_024683	[73]
	Macrosiphini	*Uroleucon erigeronense*	15,691	MZ695840	[74]

The overall lengths and A + T contents of tRNAs in three aphid mitogenomes are comparable with each other. The AT skew and GC skew of tRNAs are all positive in three species. As with all known aphid mitogenomes, *trnS1* of all three species lacks the dihydrouridine (DHU) arm. In addition, there are some tRNAs without the TΨC loop, such as *trnG*, *trnI*, *trnR*, and *trnS2* in *C. keduensis*, *trnW* in *P. panicola*, and *trnG* and *trnF* in *N. piri* (Figures S1–S3).

**Figure 2:**
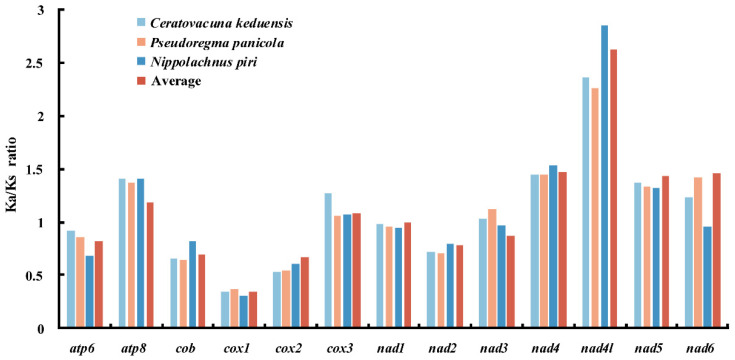
Non-synonymous/synonymous mutation (Ka/Ks) ratios of PCGs in *Ceratovacuna keduensis*, *Pseudoregma panicola* and *Nippolachnus piri* and the average Ka/Ks ratios of PCGs in all known aphid mitogenomes.

The total lengths of PCGs in *C. keduensis*, *P. panicola*, and *N. piri* are 10,935 bp, 11,028 bp, and 10,917 bp with the A + T contents of 83.80%, 83.89%, and 82.51%, respectively. PCGs of all three species show both negative AT skew and GC skew (Table 2). As in most aphids, all 13 PCGs of three species start with typical ATN codon, and terminate with TAA codon except the *cox1* in *N. piri* and *nad4* in *C. keduensis* and *N. piri*, using an incomplete T as the stop codon. The evolutionary rates of the 13 PCGs of three species are also evaluated with Ka/Ks ratios (Figure 2), and the results show that the Ka/Ks ratios of *cox1*, *cox2*, *cob*, *atp6*, *nad1*, and *nad2* in three species are lower than one, indicating these genes are under purifying selection. Almost all remaining genes show a Ka/Ks > 1, except the *nad3* and *nad6* in *N. piri* with a Ka/Ks ratio close to one. The *atp8*, *cox3*, *nad4*, *nad4l*, *nad5*, and *nad6* exhibit larger Ka/Ks ratios ranging from 1.055 of *cox3* in *P. panicola* to 2.849 of *nad4l* in *N. piri*, suggesting that these genes are under positive selections.

### 3.2. Comparative Analysis of Non-Coding Regions

**Figure 3:**
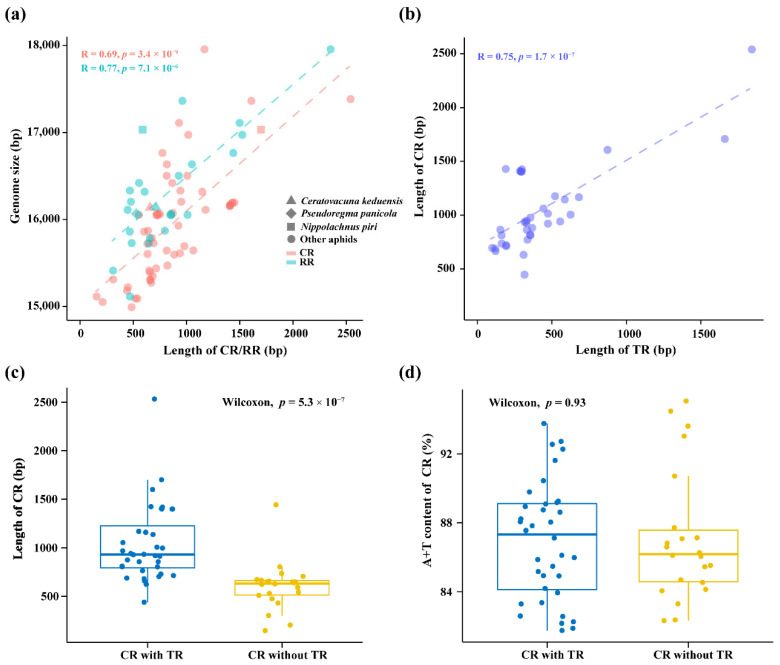
Genomic characteristics of 56 aphid mitogenomes analyzed in this study. (**a**) Correlation between mitogenome size and the length of control region (CR) or repeat region (RR). The correlations were tested using Pearson’s correlation coefficient. The same as bellow; (**b**) correlation between the lengths of control region and inserted tandem repeats (TR); (**c**) the length and (**d**) A + T content of control regions with or without tandem repeats. *p* < 0.05 denotes statistically significant difference between the two groups (Wilcoxon test).

The control region and repeat region are the two largest non-coding regions in aphid mitochondrial genomes, and the sizes of aphid mitogenomes exhibit a significant positive correlation with the lengths of both of them (Figure 3a). The non-coding control region is usually thought the most A + T rich region in aphid mitogenome and varies greatly in length from species to species, ranging from 145 bp in *Melanaphis sacchari* to 2531 bp in *M. persicae* (Table S1). The control regions in the three new mitogenomes are all located between *rrnS* and *trnI*. The control region of *C. keduensis* and *P. panicola* mitogenomes are 657 bp and 728 bp in length, respectively. In contrast, *N. piri* has a much longer control region of 1699 bp in length. In addition, tandem repeats varying in size and copy number of repeat units are identified in control regions of all three mitogenomes. A statistically significant positive correlation exists between the lengths of control regions and tandem repeats (Figure 3b), and the control regions with and without tandem repeats differ significantly in length (Figure 3c). The larger sizes of the control region and mitogenome of *N. piri* are largely attributed to the long tandem repeat sequence comprising 3.5 copies of 477 bp tandem repeat units inserted in the control region. As in the case of *P. bambucicola*, the control regions of *C. keduensis* (93.76%) and *P. panicola* (92.72%) also have higher A + T contents, which are much higher than that of *N. piri* (86.12%) in this study and most other aphids (Table 2 and Table S1). However, the control regions of three other Hormaphidinae species from Hormaphidini and Nipponaphidini have relatively lower A + T contents, which are 82.36%, 82.32%, and 83.30% in *H. spinosus*, *H. betulae*, and *S. gallarum*, respectively. There is no significant difference for A + T contents between the control regions with and without tandem repeats, in fact the control region in *P. bambucicola* with higher A + T content contains no tandem repeats (Figure 3d). These results indicate that higher A + T content in the control region may be a common feature for Cerataphidini species.

**Figure 4:**
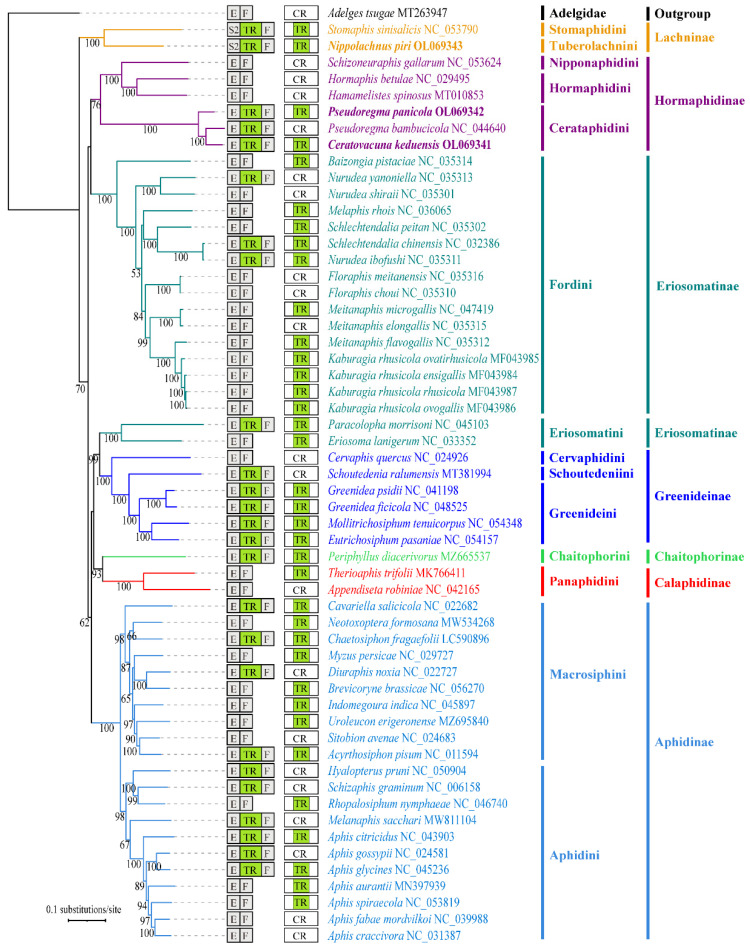
Aphid phylogeny and distribution pattern of tandem repeats in aphid mitogenomes across different clades. Bootstrap support values are indicated by numbers on nodes of phylogenetic tree, and only values > 50% are displayed. Tandem repeats (TR) include repeat sequences between *trnE* (E) and *trnF* (F) and those inserted into the control region (CR). “CR” is shown in the figure only if there is no TR in the control region. Different aphid subfamilies are indicated by different colors. Three aphid species in this study are highlighted in bold. S2, *trnS2*.

As is the case with *P. bambucicola* from the same tribe, *C. keduensis* and *P. panicola* also contain a tandem repeat region locating between *trnE* and *trnF* (Figure 4). While three species from the same subfamily Hormaphidinae but other tribes, including two Hormaphidini species (*H. spinosus* and *H. betulae*) and one Nipponaphidini specie (*S. gallarum*), do not have this tandem repeat region in their mitogenomes. The mitogenome of *N. piri* also contains a tandem repeat region, which is rearranged with the *trnF* to a new location in the downstream of *trnS2* from the ancestral position between *trnE* and *trnF*. The same pattern is found in a previously reported mitogenome of another Lachininae species (*S. sinisalicis*), which has undergone a TDRL event among the repeat region and some adjacent genes [22]. The repeat regions in each of the three newly sequenced mitogenomes differ from each other in length and copy number of tandem repeat units. The repeat region of *C. keduensis* is 715 bp in length, comprising nearly three copies of 241 bp tandem repeat units. In contrast, in *P. panicola*, the overall length of repeat region is 528 bp, consisting of two complete tandem repeat units (254 bp) and a 20 bp incomplete repeat unit. The repeat region of *N. piri* (589 bp) encompasses a 300 bp complete repeat unit and a 289 bp partial repeat unit.

Figure 4 shows the phylogenetic pattern of the tandem repeats between *trnE* and *trnF* and that inserting into the control region among different aphid subfamilies. Both show a scattered distribution pattern across different aphid lineages at different taxonomic levels. Similarities between these repeat sequences were investigated to understand the evolution of tandem repeats. Notably, the tandem repeats generally found between *trnE* and *trnF* differ greatly from those inserted into control regions in size and number of tandem repeat units among different aphid lineages (Figure 4; Table S1). There is also a low sequence similarity between these two kinds of tandem repeats. The tandem repeats within the control region of *N. piri* and *S. sinisalicis* show low sequence similarity with each other. This is also found among CR tandem repeats of four closely related Greenideini species. However, the CR tandem repeats of two Cerataphidini species (*C. keduensis* and *P. panicola*) show higher sequence similarity, and that of two Fordini species (*N. ibofushi* and *S. chinensis*) are identical. In addition, there are two extremely similar and discontinuous tandem repeats with both almost identical lengths and copy numbers of repeat units inserted into the control regions of four *Kaburagia rhusicola* subspecies and their closely related *M. flavogallis*. Similar CR repeat sequences have also been found in some Aphidinae species: for example, the control regions of *A. pisum* and *B. brassicae* contain exactly the same tandem repeats in their mitogenomes, and relatively high similarity is also detected among CR tandem repeat sequences of four *Aphis* species. The RR tandem repeats of three Cerataphidini species show high sequence similarities with each other. There is also a high-level sequence similarity between RR tandem repeats of two closely related Fordini species (*N. ibofushi* and *S. chinensis*), but both show a poor alignment with that of more distantly related species *N. yanoniella* from the same tribe. In addition, high sequence similarities are also detected among RR repeat sequences of five Greenideinae species from different tribes, and among 10 Aphidinae species from two tribes. While there is a low sequence similarity among species with relatively distant relationships, such as two Lachininae species (*S. sinisalicis* and *N. piri*) from different tribes. Thus, the distribution of both types of tandem repeats shows no clear phylogenetic patterns across or within aphid subfamilies or genus. Only some related species belonging to the same tribe show higher sequence similarity of CR tandem repeats. While for tandem repeats between *trnE* and *trnF*, it seems that most species belonging to the same subfamily have similar repeat sequences except for a few species with relatively distant relatives.

### 3.3. Gene Rearrangement of Aphid Mitogenomes

**Figure 5:**
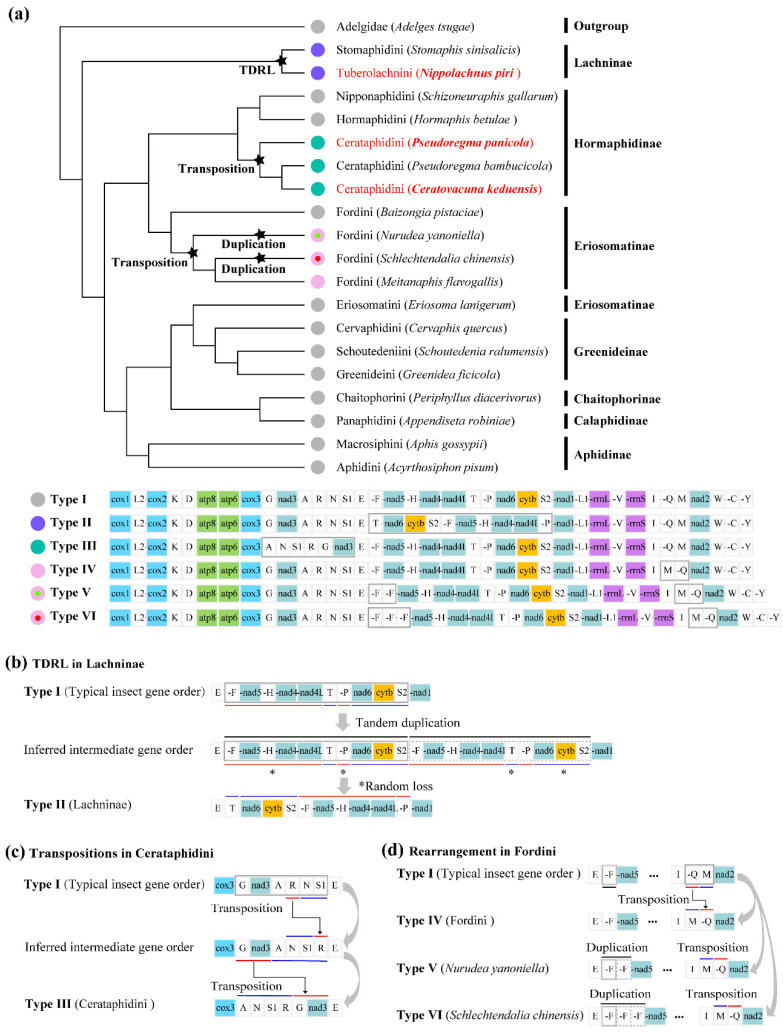
Mitogenome rearrangements across different aphid lineages. (**a**) Phylogenetic pattern of mitogenome rearrangements in aphids. Rearrangement events occurring in different species or phylogenetic clades are indicated by asterisks. Only one representative species was randomly retained in each tribe for a same gene arrangement pattern. Rearranged gene regions are shown in gray boxes; Inferred rearrangement steps for (**b**) Lachninae, (**c**) Cerataphidini, and (**d**) some Fordini mitogenomes. Rearranged gene blocks are highlighted in red and blue underlines; asterisks in (**b**) indicate random loss of genes; “…” indicates omitting genes that have not rearranged.

The gene orders of three newly determined mitogenomes were compared with that of other available aphid mitogenomes to investigate the gene rearrangement patterns across different aphid lineages. In addition to the typical insect mitogenome arrangement (Type I), there are five other types of gene arrangement in aphid mitogenomes, which are represented by 20 aphid species from three distantly related clades (Figure 5a). For three species in this study, the *N. piri* (Tuberolachnini) mitogenome exhibits a different gene arrangement pattern from *C. keduensis* and *P. panicola* and most other known aphid mitogenomes, but shows the same dramatically rearranged gene order as *S. sinisalicis*, another Lachninae species belonging to tribe Stomaphidini (Figure 5a,b). The observed rearranged gene order has been inferred to be derived from the ancestral insect gene order through a TDRL event occurred among genes between *trnE* and *nad1* [22]. This rearrangement event occurred initially by the tandem duplication of *trnF*-*nad5*-*trnH*-*nad4*-*nad4l*-*trnT*-*trnP*-*nad6*-*cob*-*trnS2*, forming an intermediate arrangement *trnF*-*nad5*-*trnH*-*nad4*-*nad4l*-*trnT*-*trnP*-*nad6*-*cob*-*trnS2*-*trnF*-*nad5*-*trnH*-*nad4*-*nad4l*-*trnT*-*trnP*-*nad6*-*cob*-*trnS2*, and followed by random loss of several redundant copies of duplicate gene blocks, including the first copy of *trnF*-*nad5*-*trnH*-*nad4*-*nad4l* and *trnP*, as well as the second copy of *trnT* and *nad6*-*cob*-*trnS2*, generating the present gene order in Lachninae (Type II) (Figure 5b).

The aphids *C. keduensis* and *P. panicola* share the same mitochondrial gene order with *P. bambucicola*, and these three Cerataphidini species exhibit a highly rearranged mitochondrial gene order (Type III) (Figure 5a,c). These gene order changes should result from two transpositions occurred within genes locating between *cox3* and *trnE*, including the transposition of *trnR* and gene block *trnN*-*trnS1* and the subsequent transposition of *trnG*-*nad3* and *trnA*-*trnN*-*trnS1*-*trnR* (Figure 5c). However, three Hormaphidinae species from other two tribes present a conserved ancestral insect gene arrangement (Figure 5a). Thus, this observed mitogenome rearrangement pattern may be a specific feature for Cerataphidini species.

Except for the two cases mentioned above, transposition of *trnQ* and *trnM* (Type IV) occurred in most Fordini species and concurrent *trnF* duplications resulted in two and three copies of *trnF* in *N. yanoniella* (Type VI) and *S. chinensis* (Type V), respectively (Figure 5a,d). Notably, during our analysis, some errors were found in mitogenome annotations released in GenBank for some Aphidinae species, which may lead to inappropriate understanding of the evolutionary patterns of aphid mitogenome rearrangements. For example, according to the original annotation, reversions exist in both ribosomal genes in *Aphis fabae mordvilkoi* mitogenome, but our systematic comparative analysis showed this is not the case. A similar situation was also found for other Aphidinae species, including *A. pisum*, *A. craccivora*, *A. gossypii*, *M. persicae*, *S. graminum* and the recently published mitogenome of *B. brassicae*, along with the outgroup of *A. tsugae*. In most cases, some tRNAs were incorrectly annotated as being reversed, transposed, lost, or duplicated. However, mitogenome re-annotation coupled with homologous sequence alignment with related species has shown that Aphidinae species show a typical gene arrangement as the putative ancestral insect gene order.

## 4. Discussion

### 4.1. Effect of Non-Coding Regions on Mitogenome Size Variation in Aphids

Our analyses show that the lengths of the non-coding control region (CR) and repeat region (RR) vary widely among mitogenomes of different aphid species, which in turn can drive major differences in mitogenome size. Aphids with larger mitogenomes usually contain longer CRs or RRs, such as the larger tandem RR in *A. glycines* (2343 bp) [63] and *S. sinisalicis* (1489 bp) [22], and the larger CR in *M. persicae* (2531 bp) [59] and *G. ficicola* (1598 bp) [55]. The CR has been thought to play important role in the transcription and replication of mitogenomes [75]. Our analyses also show that the length of CR in aphid mitogenomes depends mainly on the length variation of inserted tandem repeats. The driving force for insertion of tandem repeats within CR has been thought to probably be replication errors [76]. 

### 4.2. Phylogenetic Patterns of Tandem Repeats in Aphid Mitogenomes

The dispersed distribution pattern of RR within and among different aphid subfamilies, coupled with its presence in the basal Lachninae species (*S. sinisalicis* and *N. piri*), provides further evidence for the widely accepted hypothesis that RR likely originated from the most recent common ancestor of Aphididae and has been lost many times during subsequent species diversification [22,24,56,63]. The presence of RR in all known Cerataphidini mitogenomes (including that of two newly sequenced) but absence in Hormaphidini and Nipponaphidini indicates that this feature was once present in the most common ancestor of Hormaphidinae species, but was subsequently retained only in Cerataphidini and lost in Hormaphidini and Nipponaphidini. The RR is supposed to be another origin of the mitogenome replication by some authors [77,78], but its function is still unknown and lacks experimental evidence.

The tandem repeats inserted into the CRs also have no clear phylogenetic patterns among and within aphid subfamilies or genera. Our analyses of sequence similarity of tandem repeats within the CRs and the RRs reveal that no similarities exist between these two kinds of repeat sequences even for the same species, which may imply they have different origins during mitogenome evolution. However, separate investigations on the two respective types of tandem repeats show that some closely related species tend to have more similar CR or RR repeat sequences. Moreover, low RR sequence similarity among species with relatively distant relationships, for example two Lachininae species from different tribes, indicates that the RRs may diverge rapidly during species diversification of different aphid lineages. This also indicates that the repeat region is perhaps not suitable to be used as a molecular marker in investigating the evolutionary history of aphid lineages at higher taxonomic levels.

### 4.3. Evolution of Gene Rearrangement in Aphid Mitogenomes

Gene rearrangements can be used as genome “morphology” in phylogenetic inference and are thought to be important molecular markers for uncovering insect evolution [16,79]. Based on current mitogenome data, our study summarizes six types of gene arrangement patterns, with most species exhibiting a conserved ancestral insect gene order. Gene rearrangements are only found in phylogenetically related species within three clades at different taxonomic levels, which may have evolved from the ancestral gene order through independent evolutionary events. In the case of Fordini, transposition of *trnQ* and *trnM* occurred in all species in this tribe except the basal species *B. pistaciae*, leading to the speculation that this gene rearrangement occurred in the most recent common ancestor of these species only after its divergence from the ancestor of *B. pistaciae*. Concurrent *trnF* duplications also contribute mitogenome rearrangements of Fordini species. Two remarkable rearrangement types are found in species from Cerataphidini and Lachninae, respectively. Cerataphidini, along with other two tribes Hormaphidini and Nipponaphidini, belong to the subfamily Hormaphidinae [80,81]. All three Cerataphidini mitogenomes (including that of two newly sequenced) are found to be highly rearranged with the same pattern, while mitogenomes of the other two tribes exhibit an ancestral insect gene order, indicating that this rearrangement is probably a Cerataphidini-specific event and may have occurred independently in the most recent common ancestor of Cerataphidini. Though the exactly cause of mitogenome rearrangements is not yet elucidated, life-history traits are considered to be important influencing factors for rearrangements, for example, the parasitism has been suggested to be a key inducer of higher rearrangement rates in mitogenomes of some hymenopteran parasitoids [16,82]. Cerataphidini has relatively distant phylogenetic relationships and different biological characteristics (e.g., host association) with the other two tribes [80,81]. It is uncertain whether the different mitogenome arrangement patterns between Cerataphidini and the other two tribes are associated with host association. However, the gene arrangement patterns of three hormaphidine tribes provide further evidence to corroborate current view of phylogenetic relationships among the three aphid tribes, and further demonstrate the value of gene rearrangement patterns in studying the evolutionary relationships among insect lineages.

An extraordinary mitogenome rearrangement involving five PCGs, five tRNAs, and the RR caused by TDRL event is observed in mitogenomes of two species (including the newly sequenced *N. piri* mitogenome) from different tribes of Lachninae, which represents a particular aphid lineage comprising various clades feeding on both conifers and broad-leaved plants. This particular rearrangement pattern further indicates that this gene arrangement is at least a common feature derived from the most common ancestor of Stomaphidini + Tuberolachnini, and even probably a common feature for all Lachninae species. Considering Lachninae includes other tribes in addition to Stomaphidini and Tuberolachnini [83], additional new mitogenomes from those clades are sorely needed in future work to address whether Lachninae species share the same mitogenome rearrangement pattern.

## 5. Conclusions

This paper reports three new complete mitogenomes of Hormaphidinae and Lachninae species. All three mitogenomes contain a tandem repeat region normally between *trnE* and *trnF* and show notably rearranged gene orders. Along with a previously published Cerataphidini mitogenome, the two new mitogenomes of two Cerataphidini species, *C. keduensis* and *P. panicola*, share the same rearranged gene order, but differ from that of the other two Hormaphidinae tribes; in addition, mitogenomes of Cerataphidini species also contain a special tandem repeat region and a control region with much higher A + T content, indicating that Cerataphidini may have experienced unique history of mitogenome evolution. The mitogenomes of two Lachninae species, *N. piri* (Tuberolachnini) and *Stomaphis sinisalicis* (Stomaphidini), have a same large-scale gene rearrangement pattern, which may suggest that this feature is derived from at least the common ancestor of Stomaphidini + Tuberolachnini or even the ancestor of Lachninae. Our analyses also provide further evidence for the hypothesis that the repeat region likely originated from the most recent common ancestor of aphids. Overall, our study provides new insights into the evolution of gene rearrangement and repeat region in aphid mitogenomes, and the obtained mitogenomes provide important data resources for future comparative studies.

## Figures and Tables

**Table 2:** Base composition of *Ceratovacuna keduensis*, *Pseudoregma panicola* and *Nippolachnus piri* mitogenomes.

Regions	Size (bp)	Nucleotides Composition (%)						AT-Skew	GC-Skew
		T	C	A	G	A + T	G + C		
Full genome									
*C. keduensis*	16,138	39.84	9.64	45.15	5.36	84.99	15.00	0.062	−0.285
*P. panicola*	16,059	39.82	9.66	45.15	5.37	84.97	15.03	0.063	−0.285
*N. piri*	17,033	37.67	10.53	46.22	5.58	83.89	16.11	0.102	−0.307
PCGs ^1^									
*C. keduensis*	10,935	48.42	8.65	35.38	7.54	83.80	16.20	−0.156	−0.068
*P. panicola*	11,028	48.82	8.37	35.07	7.74	83.89	16.11	−0.164	−0.039
*N. piri*	10,917	47.16	9.21	35.36	8.27	82.51	17.49	−0.143	−0.054
1st codon									
*C. keduensis*	3645	40.47	8.70	40.36	10.48	80.82	19.18	−0.001	0.093
*P. panicola*	3676	40.72	8.79	39.96	10.53	80.69	19.31	−0.009	0.090
*N. piri*	3639	39.19	8.71	41.08	11.02	80.27	19.73	0.024	0.117
2nd codon									
*C. keduensis*	3645	53.58	13.31	22.66	10.45	76.24	23.76	−0.406	−0.120
*P. panicola*	3676	53.67	13.00	22.96	10.36	76.63	23.37	−0.401	−0.113
*N. piri*	3639	52.98	13.49	22.95	10.58	75.93	24.07	−0.396	−0.121
3rd codon									
*C. keduensis*	3645	51.22	3.95	43.13	1.70	94.35	5.65	−0.086	−0.398
*P. panicola*	3676	52.07	3.32	42.27	2.34	94.34	5.66	−0.104	−0.173
*N. piri*	3639	49.30	5.44	42.04	3.22	91.34	8.66	−0.079	−0.257
tRNAs									
*C. keduensis*	1466	42.02	5.59	44.41	7.98	86.43	13.57	0.028	0.176
*P. panicola*	1481	41.93	5.74	44.23	8.10	86.16	13.84	0.027	0.171
*N. piri*	1486	41.05	5.52	45.63	7.81	86.68	13.32	0.053	0.172
rRNAs									
*C. keduensis*	2035	44.62	4.77	40.93	9.68	85.55	14.45	0.014	0.340
*P. panicola*	2033	44.61	4.82	41.12	9.44	85.74	14.26	−0.041	0.246
*N. piri*	2075	45.59	4.72	39.08	10.60	84.67	15.33	−0.077	0.384
Repeat region									
*C. keduensis*	715	43.22	10.91	42.10	3.78	85.31	14.69	−0.013	−0.486
*P. panicola*	528	43.37	11.93	40.91	3.79	84.28	15.72	−0.029	−0.518
*N. piri*	589	32.26	10.53	55.52	1.70	87.78	12.22	0.265	−0.722
Control region									
*C. keduensis*	657	47.95	3.50	45.81	2.74	93.76	6.24	−0.023	−0.122
*P. panicola*	728	47.66	3.98	45.05	3.30	92.72	7.28	−0.028	−0.094
*N. piri*	1699	40.14	7.53	45.97	6.36	86.11	13.89	0.068	−0.085

^1^ Stop codons are excluded from the statistics of protein-coding genes.

## Data Availability

The data underlying this article are available in the GenBank Nucleotide Database at https://www.ncbi.nlm.nih.gov/genbank/, and can be accessed with accession numbers OL069341, OL069342 and OL069343.

## Supplementary Materials

The following supporting information can be downloaded at: https://www.mdpi.com/article/10.3390/ani12151970/s1. Figure S1: Secondary structures of the transfer RNA genes (tRNAs) in mitogenome of *Ceratovacuna keduensis*. Figure S2: Secondary structures of the transfer RNA genes (tRNAs) in mitogenome of *Pseudoregma panicola*. Figure S3: Secondary structures of the transfer RNA genes (tRNAs) in mitogenome of *Nippolachnus piri*. Table S1: Information of complete mitogenomes in aphids. Table S2: Features of the mitochondrial genome of *Ceratovacuna keduensis*. Table S3: Features of the mitochondrial genome of *Pseudoregma panicola*. Table S4: Features of the mitochondrial genome of *Nippolachnus piri*.
