# Supplementary material for: Insights into the Evolution of Aphid Mitogenome Features from New Data and Comparative Analysis
Source: Animals (Basel). 2022 Aug 3;12(15):1970. doi: 10.3390/ani12151970 (PMC9367533; doi:10.3390/ani12151970)
Supplement: Supplementary file 1 [file animals-12-01970-s001.zip › Supplementary_Figures.pdf]

Supplementary Figures

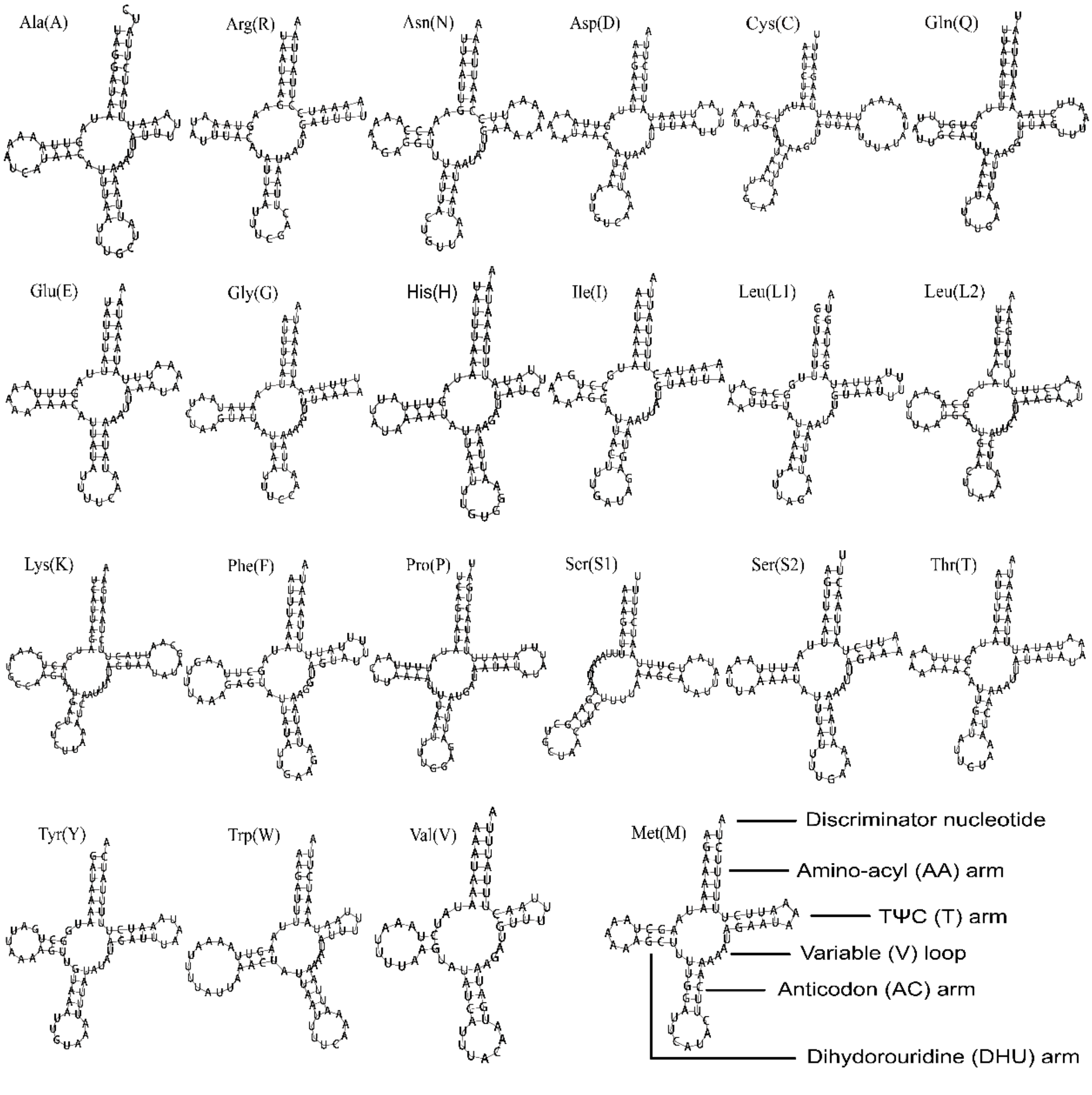

Figure S1. Secondary structures of the transfer RNA genes (tRNAs) in mitogenome of *Ceratovacuna keduensis*.

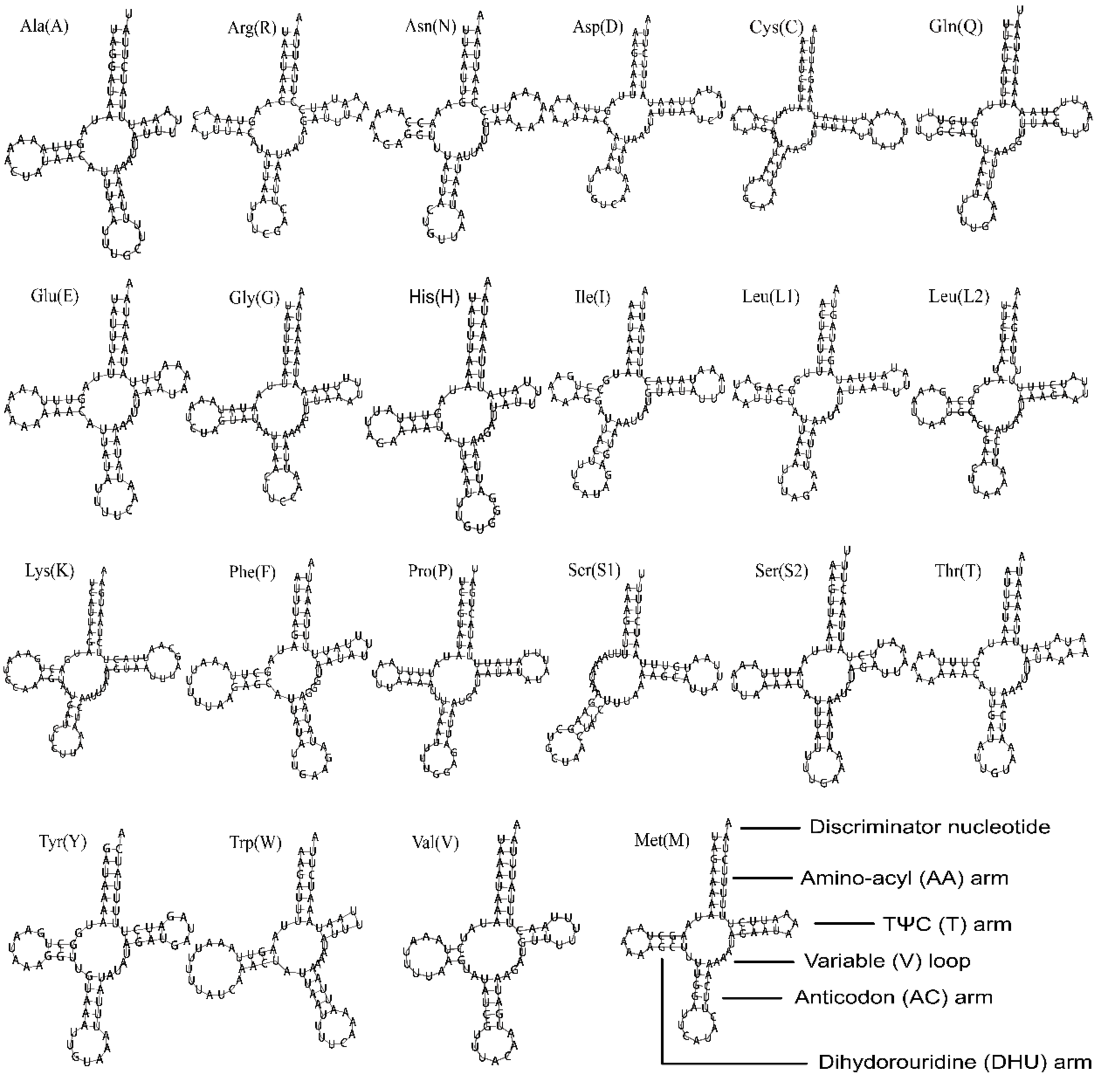

Figure S2. Secondary structures of the transfer RNA genes (tRNAs) in mitogenome of *Pseudoregma panicola*.

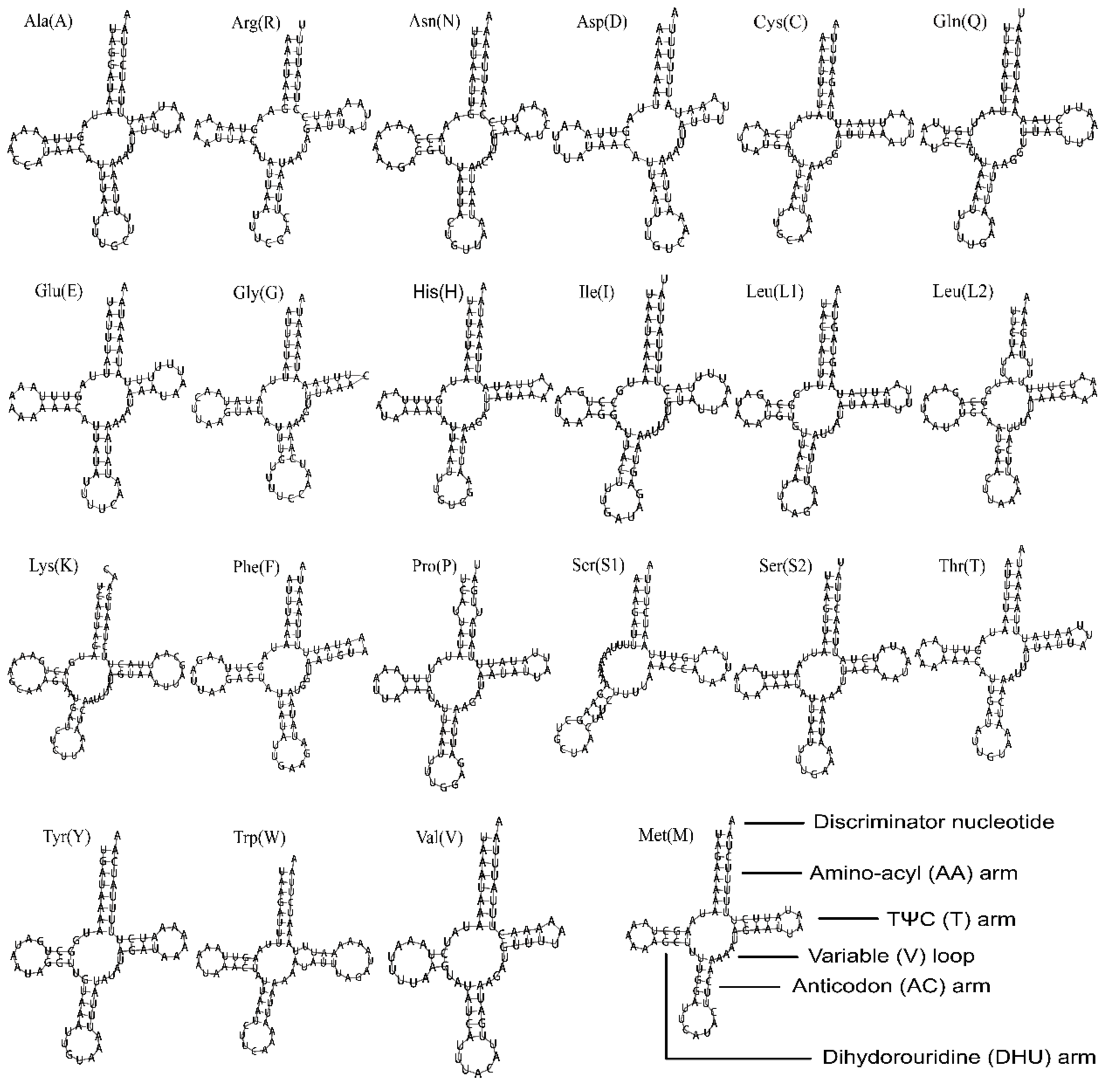

Figure S3. Secondary structures of the transfer RNA genes (tRNAs) in mitogenome of *Nippolachnus piri*.
